# Comparison of Cardiac Risk Scores among the East Mediterranean and South Asian Population

**DOI:** 10.4314/ejhs.v32i1.8

**Published:** 2022-01

**Authors:** Tahani Saud Samar Alenazi, Alanoud Abdullah Slamah Alhuiti, Palanisamy Amirthalingam, Ahmed Mohsen Hamdan, Osama Salih Mohammed, Mostafa A Sayed Ali

**Affiliations:** 1 Department of Pharmacy Practice, Faculty of Pharmacy, University of Tabuk, Tabuk, Saudi Arabia; 2 Department of Internal Medicine, King Fahd Specialty Hospital, Tabuk, Saudi Arabia; 3 Department of Clinical Pharmacy, Assuit University, Egypt

**Keywords:** Cardiovascular disease, East Mediterranean, South Asian

## Abstract

**Background:**

Cardiovascular disease (CVD) is a global burden particularly in developing countries necessitates the periodical monitoring for these vulnerable population. This study aimed to compare four tools to measure the CVD risk between the East Mediterranean and South Asian population.

**Methods:**

This retrospective analysis included 139 patients from East Mediterranean (n=90) and South Asians (n=49) ethnicity who were admitted during the one-year period in a multi-specialty tertiary care hospital located in Tabuk, Saudi Arabia. Four different tools currently in use across the world were used to analyses the CVD risk.

**Results:**

Atherosclerotic Cardiovascular Disease (ASCVD) from American College of Cardiology/American Heart Association (ACC/AHA) was found to be significant (P=0.0000) than World Health Organization/International Society of Hypertension (WHO/ISH) and Framingham Risk Score (FRS) European SCORE (Systematic Coronary Risk Evaluation) memo card from European Society of Cardiology risk prediction charts. Meanwhile, FRS looks equally good as it detects 44.89% of South Asian study population with >10% CVD risk while ACC/AHA detects 46.93%.

**Conclusion:**

The present study recommends ACC/AHA cardiac risk estimator to identify the CVD risk in East Mediterranean population. However, the South Asian population needed a population-based tool to assess the accurate CVD risk.

## Introduction

Myocardial infarction is one of the leading causes of deaths, and the incidence rate is about 32.4 million per year across the world (1), with a high proportion of mortality rate reported in East Mediterranean (EM) and South Asian (SA) countries (2). Various tools have been developed to measure 10-years' cardiovascular risk; however, the usefulness is yet to be established in developing countries. The report from the recent cross-sectional surveys emphasizes the usefulness of Atherosclerotic Cardiovascular Disease (ASCVD) from the American College of Cardiology/American Heart Association (ACC/AHA) (3), World Health Organization/International Society of Hypertension (WHO/ISH) (4) and Framingham Risk Score (FRS) for hard coronary heart disease from the Framingham heart study (5) among the Saudi (6,7,8) and SA populations (9,10). Saudi Arabia has a population of around 34 million as of 2020 (11), with 38% expatriates, especially from other parts of the EM region and SA countries (12,13). However, these cross-sectional surveys were conducted in the Jeddah City and the Western, Eastern, and Northern regions (6,7,8). To the best of our knowledge, the usefulness of the abovementioned cardiac risk scores' comparison among the EM and SA populations in Saudi Arabia is yet to be established. Henceforth, this study aimed to compare the ACC/AHA, WHO/ISH, FRS, and European SCORE (Systematic Coronary Risk Evaluation) memo card from the European Society of Cardiology risk prediction charts (14) among the population in the Northern-West region of Saudi Arabia, especially between the EM and SA population.

## Patients and Methods

**Study site and population**: During the period of August 2019 to July 2020, 139 patients were admitted and diagnosed with myocardial infarction in a multi-specialty tertiary care hospital, in Tabuk, Saudi Arabia. The study was approved by the Institutional Review Board, Ministry of Health, Tabuk, Saudi Arabia. This retrospective analysis included 139 patients between the ages of 40–70 years, and those categorized in the East Mediterranean (n=90) population belonged to Saudi Arabia, Egypt, Jordan, Sudan, Yemen, and Kuwait. The remaining study population (n=49) belonged to South Asia, including India, Pakistan, Bangladesh and Sri Lanka.

**Data collection**: A comprehensive data collection was conducted at the Medical Record Department (MRD), and these data included demographic variables (age, gender, nationality, social history, past medical history, past medication history, family history etc.), diagnoses (STEMI and NSTEMI), and cardiometabolic parameters (systolic blood pressure (SBP) and diastolic blood pressure (DBP)), fasting blood sugar (FBS), total cholesterol (TC), triglycerides (TGs), low-density lipoproteins (LDL), and high-density lipoproteins (HDL).

**Cardiac risk scores**: Four 10-year risks for cardiac risk score calculators/charts included in this study were ASCVD (ACC/AHA), FRS, SCORE, and WHO/ISH risk prediction charts. Comparison of clinical and biochemical parameters included in the cardiovascular risk calculators/charts was illustrated in Table 1. To unify the 10-years, the following adjustments were made in order to facilitate the data input for all four cardiac scores; further, the possible range was commonly used, such as age in years (40–70 years), SBP (120–180 mmHg) and TC (4–8 mmol/L).

**Table 1:** Clinical and bio-chemical parameters included in cardiac risk calculators/charts

Parameters	ASCVD	FRS	SCORE	WHO/ISH
Age	✓ (40–79 years)	✓ (30–79 years)	✓ (40–-70 years)	✓ (40–79 years)
Gender	✓	✓	✓	✓
Ethnicity	✓	✗	✓	✓
SBP (mmHg)	✓	✓	✓	✓
DBP (mmHg)	✓	✗	✗	✗
TC (mmol/L)	✓	✓	✓	✓
HDL (mmol/L)	✓	✗	✗	✗
LDL (mmol/L)	✓	✗	✗	✗
Smoking history	✓	✓	✓	✓
History of diabetes	✓	✗	✗	✓
On hypertension treatment	✓	✓	✗	✗
On statin treatment	✓	✗	✗	✗
On aspirin treatment	✓	✗	✗	✗

**Data analysis**: Chi-squared test was used to analyze the association of demographic variables, clinical variables, and distribution of cardiac risk scores. Unpaired ‘t’ test was used to compare the mean ± standard deviation (SD) between the EM and SA populations. Statistical Package for Social Sciences (SPSS) version 25 was used in the statistical analysis, and P-value < 0.05 was considered as statistically significant.

## Results

**Association of demographic and clinical variables among the study population**: Table 2 represents the comparison of demographic and clinical variables between the study population. The majority of both EM and SA populations were males. Each of the 38.84% belonged to the 40–49 years and 50–59 years with 40–59 years of age; however, 40–49 years of age in EM (40%) and 50–59 years of age (48.97%) in the SA population were predominant; however, there was no statistical association between them. Similarly, there was no significant association on the smoking history (P=0.0503), even though the number of smokers was high in the EM population; meanwhile, non-smokers were more in the SA population. Most of them had past medical history, including diabetes mellitus, hypertension, and NSTEMI irrespective of ethnicity; there was no significant association between the EM and SA population in regard to past medical history (P=0.3467) and type of infarction (P=1.0000).

**Table 2:** Association of demographic and clinical variables among the study population

Parameters	Total study population (139) n (%)	Eastern Mediterranean population (90) n (%)	South Asian population (49) n (%)	P value*
Age in years				
40 – 49 years	54 (38.84)	36 (40)	18 (36.73)	
50 – 59 years	54 (38.84)	30(33.33)	24 (48.97)	0.37
60 – 69 years	31 (22.29)	24 (26.66)	9 (14.28)	
Gender				
Male	124 (89.20)	79 (87.77)	45 (91.83)	0.57
Female	15 (10.79)	11 (12.22)	4(8.16)	
Current smokers				
Yes	68 (48.92)	50 (55.55)	18 (36.73)	0.05
No	71 (51.07)	40 (44.44)	31 (63.26)	
Past medical history				
Yes	93 (66.90)	63 (70)	30 (61.22)	0.34
No	46 (33.09)	27 (30)	19 (38.77)	
Type of infarction				
STEMI	63 (45.32)	41 (45.55)	22 (44.89)	1.00
NSTEMI	76 (54.67)	49 (54.44)	27(55.10)	

* Chi-square test among the between Eastern Mediterranean population and South Asian population

P<0.05 considered statistically significant

**Comparison of cardiometabolic parameters among the study population**: Mean±SD of cardiometabolic parameters was observed among the study population (Table 3). SBP was borderline high in total population (135.29±27.25) and EM (134.90±28.69) and SA populations (136±24.66); on the other hand, mean±SD of DBP was found to be normal irrespective of the ethnicity. Fasting blood sugar was high among the total study population; particularly, the mean±SD of FBS was higher in EM than SA; this association has no statistical significance (P=0.2004). Mean±SD of TC was found to be normal; however, TGs and LDL levels were elevated with the decline in HDL level in the total study population. Additionally, no statistical significance was observed despite TGs (P=0.0677) and LDL (P=0.4293) being high in the SA than EM population.

**Table 3:** Comparison of cardio metabolic parameters among the study population

Parameters	Total study population (139) Mean (SD)	Eastern Mediterranean population (90) Mean (SD)	South Asian population (49) Mean (SD)	P value*
Systolic blood pressure (mmHg)	135.29 (27.25)	134.90 (28.69)	136 (24.66)	0.82
Diastolic blood pressure (mmHg)	80.49 (15.53)	80.19 (13.88)	81.04 (18.33)	0.75
Fasting blood sugar (mmol/L)	10.28 (6.03)	10.75 (6.38)	9.36 (5.24)	0.20
Total cholesterol (mmol/L)	4.43 (1.28)	4.28 (1.21)	4.70 (1.37)	0.06
Triglycerides (mmol/L)	1.80 (1.09)	1.67 (1.01)	2.03 (1.18)	0.06
LDL (mmol/L)	2.81 (1.19)	2.75 (1.09)	2.92 (1.35)	0.42
HDL (mmol/L)	0.94 (0.32)	0.94 (0.38)	0.93 (0.18)	0.82

* Unpaired Student's ‘t’ test between Eastern Mediterranean population and South Asian population

P<0.05 considered statistically significant

**Comparison of various cardiac risk scores among the study population**: Table 4 denotes the sensitivity of detecting cardiovascular risk score among the study population as ACC/AHA> FRS> WHO/ISH> SCORE. The ACC/AHA and FRS had a significant association (P=0.0000) with detecting myocardial infarction, where SCORE and WHO/ISH cardiac scores were mostly detected as <10% (SCORE: 91.36%; WHO/ISH: 62.58%) among the total study population. Among the ACC/AHA and FRS, the ACC/AHA has been detected in the majority of the total study population (51.79%) and EM population (54.44%) with >10% risk; however, ACC/AHA managed to be detected >10% in only 46.93% of the SA population.

**Table 4:** Comparison of various cardiac risk scores among the study population

CVD risk	Total population (139)				Eastern Mediterranean population (90)				South Asian population (49)			

	ACC/A HA n (%)	FRS n (%)	SCOR E n (%)	WHO/IS H n (%)	ACC/A HA n (%)	FRS n (%)	SCOR E n (%)	WHO/IS H n (%)	ACC/A HA n (%)	FRS n (%)	SCOR E n (%)	WHO/IS H n (%)
< 10%	67 (48.20)	73 (52.51)	127 (91.36)	87 (62.58)	41 (45.55)	46 (51.11)	79 (87.77)	56 (62.22)	26 (53.06)	27 (55.10)	48 (97.95)	31 (63.26)
> 10%	72 (51.79)	66 (47.48)	12 (8.63)	52 (37.41)	49 (54.44)	44 (48.88)	11 (12.22)	34 (37.77)	23 (46.93)	22 (44.89)	1 (2.04)	18 (36.73)
P value*	**0.00**			**0.00**				**0.00**				

* Chi-square test among the Eastern Mediterranean population and South Asian population

P<0.05 considered statistically significant

## Discussion

This study compared the applicability and predictability of four risk assessment instruments to detect cardiovascular diseases in a sample of East Mediterranean and South Asian populations. The present study showed that the ACC/AHA cardiac risk prediction score was more useful in detecting the 10-year ASCVD risk. The ACC/AHA instrument identified more than half of the study population, especially the East Mediterranean population, at high ASCVD risk (>10%) followed by FRS, WHO/ISH, and SCORE, and this result substantiates the recent findings [6] reported from Saudi Arabia. The ACC/AHA cardiac risk estimator incorporates unique clinical parameters (HDL, LDL), which could be the reason for more accuracy in detecting cardiac risk. In this context, HDL acts as anti-inflammatory (17), antioxidant (18), anti-apoptotic (19), and anti-thrombotic (20) towards protecting from cardiovascular disease (CVD) risk; hence, the decreased level of HDL in our study population could be a potential reason for the accuracy of ACC/AHA cardiac risk score. Meanwhile, the raised LDL in our study population and its impact on CVD risk (21) may be a contributor for improved accuracy.

Smoking is a major risk factor that doubles the incidence of CVD risk (22), which is substantiated in our study, as almost one half of the overall study population and the majority of EM population were current smokers. On the contrary, the majority of SA population were non-smokers; as such, other causes such as changes in lifestyle (23) and mental health issues (24, 25) are more likely to be the risk factors for developing CVD among the SA population (26), especially expatriates (27). Also, it should be noted that TC, TG, and LDL were comparatively elevated in the SA population than in the EM population and this finding was already well established in the past (28).

Although the ACC/AHA cardiac risk score calculator is found to be better in our study population, the FRS-CVD risk assessment model has detected many cases >10% of CVD risk of 44.89% in the SA population; nevertheless, it is almost close to the ACC/AHA cardiac risk score (46.93%). The accuracy in assessing the CVD risk in the SA population remains a challenge due to the variation in CVD risk score observed in the previous study (29); hence, CVD risk assessment methods should account for the culture, environmental features, dietary traditions and linguistics (30) linked to the complexity of genetic variation in the SA population (31). Consequently, our result is consistent with the previous studies emphasizing the need of population-specific CVD risk assessment tools required for the South Asian population (29,30).

On the other hand, EM countries have similarities in culture (32) and also have similarity in risk factors for CVD (33) such as smoking habits, physical activity, and dietary preferences (34). This offers the recommendation of ACC/AHA cardiac risk score as a unique tool to assess the CVD risk score for the EM population. Meanwhile, the report of this study recommends that further study is warranted to enlighten the suitable CVD risk score for the SA population.

Overall, the SA and the EM populations have a greater risk of developing CVD risk (35). Therefore, the present study emphasizes a periodical CVD risk assessment among these populations with Type 2 diabetes mellitus and hypertension. Although the present study has a limited study population and study period, it has pioneered the comparison of CVD risk assessment tools among the SA and EM populations. Further, the study has warranted longitudinal studies to substantiate the present findings in the near future.

This study recommends the ACC/AHA cardiovascular risk estimator, which demonstrates higher detection of CVD risk for the East Mediterranean population. However, the accurate risk assessment of the South Asian population needs a standardized tool to account for the diversity in culture, lifestyle and traditional beliefs.
